# Regular consumption of vitamin D-fortified yogurt drink (*Doogh*) improved endothelial biomarkers in subjects with type 2 diabetes: a randomized double-blind clinical trial

**DOI:** 10.1186/1741-7015-9-125

**Published:** 2011-11-24

**Authors:** Sakineh Shab-Bidar, Tirang R Neyestani, Abolghassem Djazayery, Mohammad-Reza Eshraghian, Anahita Houshiarrad, A'azam Gharavi, Ali Kalayi, Nastaran Shariatzadeh, Malihe Zahedirad, Niloufar Khalaji, Homa Haidari

**Affiliations:** 1 Department of Nutrition and Biochemistry, School of Public Health, Tehran University of Medical Sciences, Tehran, Iran; 2 Department of Nutrition Research, National Nutrition and Food Technology Research Institute and Faculty of Nutrition and food Technology, Shahid Beheshti University of Medical Sciences, Tehran, Iran; 3 Department of Biostatistics and Epidemiology, School of Public Health, Tehran University of Medical Sciences, Tehran, Iran

## Abstract

**Background:**

Endothelial dysfunction has been proposed as the underlying cause of diabetic angiopathy that eventually leads to cardiovascular disease, the major cause of death in diabetes. We recently demonstrated the ameliorating effect of regular vitamin D intake on the glycemic status of patients with type 2 diabetes (T2D). In this study, the effects of improvement of vitamin D status on glycemic status, lipid profile and endothelial biomarkers in T2D subjects were investigated.

**Methods:**

Subjects with T2D were randomly allocated to one of the two groups to receive either plain yogurt drink (PYD; containing 170 mg calcium and no vitamin D/250 mL, n_1 _= 50) or vitamin D3-fortified yogurt drink (FYD; containing 170 mg calcium and 500 IU/250 mL, n_2 _= 50) twice a day for 12 weeks. Anthropometric measures, glycemic status, lipid profile, body fat mass (FM) and endothelial biomarkers including serum endothelin-1, E-selectin and matrix metalloproteinase (MMP)-9 were evaluated at the beginning and after the 12-week intervention period.

**Results:**

The intervention resulted in a significant improvement in fasting glucose, the Quantitative Insulin Check Index (QUICKI), glycated hemoglobin (HbA1c), triacylglycerols, high-density lipoprotein cholesterol (HDL-C), endothelin-1, E-selectin and MMP-9 in FYD compared to PYD (*P *< 0.05, for all). Interestingly, difference in changes of endothelin-1, E-selectin and MMP-9 concentrations in FYD compared to PYD (-0.35 ± 0.63 versus -0.03 ± 0.55, *P *= 0.028; -3.8 ± 7.3 versus 0.95 ± 8.3, *P *= 0.003 and -2.3 ± 3.7 versus 0.44 ± 7.1 ng/mL, respectively, *P *< 0.05 for all), even after controlling for changes of QUICKI, FM and waist circumference, remained significant for endothelin-1 and MMP-9 (*P *= 0.009 and *P *= 0.005, respectively) but disappeared for E-selectin (*P *= 0.092). On the contrary, after controlling for serum 25(OH)D, the differences disappeared for endothelin-1(*P *= 0.066) and MMP-9 (*P *= 0.277) but still remained significant for E-selectin (*P *= 0.011).

**Conclusions:**

Ameliorated vitamin D status was accompanied by improved glycemic status, lipid profile and endothelial biomarkers in T2D subjects. Our findings suggest both direct and indirect ameliorating effects of vitamin D on the endothelial biomarkers.

**Trial registration:**

ClinicalTrials.gov: NCT01236846

## Background

The prevalence of type 2 diabetes (T2D) is increasing worldwide, including Iran [1]. It has been shown that diabetes is accompanied by remarkably greater risk for cardiovascular disease (CVD). Accounting for more than 80% of all premature deaths, CVD has been known as the major cause of mortality in T2D [2].

Diabetes may affect both small and large vessels, leading to micro- and macro-angiopathy, respectively. Hyperinsulinemia and augmented oxidative stress, usually both present in diabetes, are known as two major contributing factors of the long-term complications, including micro- and macro-angiopathy [3]. It is hypothesized that endothelial dysfunction is the underlying cause of diabetic angiopathy that eventually leads to CVD [4]. However, a population-based study showed that raised plasma concentrations of endothelial biomarkers may predict diabetes independent of such diabetes risk factors as obesity, insulin resistance and systemic inflammation [5]. The endothelial function may therefore be the focus of preventive efforts against both diabetes and its fatal complications.

The relationship between vitamin D and T2D has recently been the focus of interest. Vitamin D, mostly known for its calcemic functions, has been shown to have many non-calcemic actions including regulation of gene expression and antioxidant properties [6]. Vitamin D deficiency has been proposed as an independent risk factor for CVD [7]. It has been recently reported that the odds ratio for having CVD outcomes in individuals with serum 25(OH)D below 25 nmol/L, compared to those with 25(OH)D ≥37.5 nmol/L, after adjustment for potential confounders was 2.90 (95% confidence interval: 1.67 to 5.12, *P *< 0.001) [8]. Considering the role of endothelial dysfunction in development of CVD, the issue has been raised if vitamin D can influence the endothelia. Vitamin D deficiency has been associated with endothelial dysfunction and lipid peroxidation in non-diabetic adults [9]. We have recently shown in another study on a separate population that vitamin D intake in subjects with T2D improves glycemic control [10]. In the current study, it was hypothesized that the effect of improvement of vitamin D status via daily intake of a vitamin D3-fortified Persian yogurt drink (doogh) can affect endothelial biomarkers independent of glycemic status.

## Methods

### Sample size

Based on our previous data on the mean serum 25(OH)D in Iranian diabetic patients (57.8 ± 47.8 nmol/L) [11], to detect a change in mean 25(OH)D of 1 standard deviation (SD; effect size of 1) and to have a power of 90%, the calculated sample size was 50 in each group.

### Subjects

A total of 100 known patients with T2D (57 women and 43 men) 52.5 ± 7.4 years old (range 29 to 67 years) were randomly selected from our original study population [12], all recruited from the Iranian Diabetes Society or Gabric Diabetes Society, both located in Tehran. The inclusion criteria were: (a) age 25 to 70 years old, (b) willingness to participate, and (c) no use of vitamin, dietary, herbal or omega-3 supplements for at least 3 months prior to the intervention period. Exclusion criteria included: (a) history of cardiovascular, gastrointestinal, renal, and other endocrinological diseases, (b) pregnancy or lactation, (c) receiving insulin, and (d) treatment for weight reduction. All subjects were already receiving oral hypoglycemic medications including metfromin, glibenclamide and glitazone. There was no statistical difference between the two groups in distribution of each drug usage (data not shown).

### Study protocol

**Figure 1:**
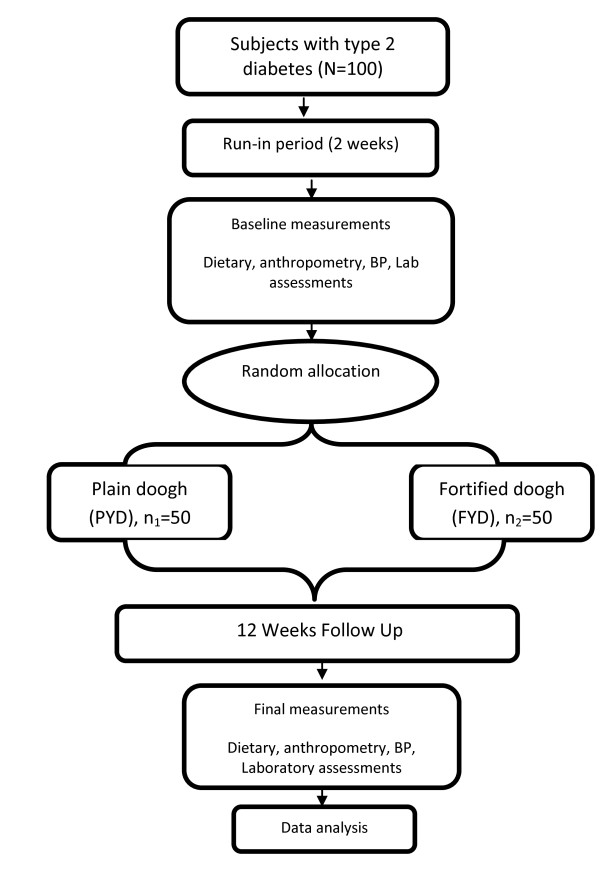
**The study protocol at a glance**.

Figure 1 shows the study protocol. The study was a part of a bigger research project on the evaluation of the effect of vitamin D receptor variants on response to vitamin D intake [12]. This was a 3-month randomized controlled clinical trial (RCT) bicentrally conducted during mid-October 2010 to late March 2011 by the National Nutrition and Food Technology Research Institute (NNFTRI) and School of Public Health of Tehran University of Medical Sciences (TUMS). On the first visit, the study protocol and objectives were fully explained to all participants before they were given an informed written consent to sign. After a 2-week run-in period, participants were randomly allocated to either vitamin D3-fortified doogh (FYD; containing 170 mg calcium and 500 IU vitamin D3/250 mL; n_1 _= 50) or plain doogh (PYD; containing 170 mg calcium and no detectable vitamin D/250 mL; n_2 _= 50). Protein, fat and energy contents of dooghs were 1.4 g/dL, 1.0 g/dL and 30 kcal/dL, respectively. Participants were instructed to consume a bottle of doogh with both lunch and dinner, that is 500 mL/day equaling 1000 IU vitamin D3 a day in FYD group. The intervention period was 12 weeks. Consumption of 1000 IU/day vitamin D is believed to be a safe and effective way to increase circulating 25(OH)D in this study period [10]. Participants were given dooghs in 30-bottle packs, which were enough for 2 weeks. All subjects were visited biweekly both to assess their compliance and to provide them with dooghs for the next two weeks. Blood pressure, anthropometric, dietary, body fat and laboratory evaluations were preformed both before and after the intervention. Both participants and examiners were unaware of the group allocations. The primary outcome was considered a significant rise in serum 25(OH)D3 in the FYD group. The study protocol was approved scientifically and ethically by appropriate committees of NNFTRI, Shahid Beheshti University of Medical Science, and TUMS.

### Assessment of dietary intake

Dietary intake was assessed using a 24-hour recall questionnaire for 3 days (including a weekend) at the beginning and in the end of the intervention period. All the questionnaires were completed with the aid of a trained dietitian. A food album was used to help the participants to recall the portion sizes of the foods consumed, which were then converted to grams using household measures. Data on energy and nutrient intakes were derived from food intake questionnaires using US Department of Agriculture (USDA) food composition tables with some modifications for Iranian foods.

### Anthropometrics and blood pressure

Weight was measured with light clothes and without shoes using a digital scale (Seca 808, Hamburg, Germany) to the nearest of 0.1 kg. Height was measured without shoes using a stadiometer (Seca, Hamburg, Germany) to the nearest of 0.1 cm. Circumferences of the waist and hip were evaluated by a measuring tape to the nearest of 0.1 cm. Body mass index (BMI) was calculated using the equation BMI = weight (kg)/height^2 ^(m). To measure blood pressure (BP), subjects remained in a sitting position for at least 10 minutes. BP was then measured twice by a digital sphygmomanometer (BC08, Beurer, Germany). The average of the two measurements was considered as the BP of the patient.

### Laboratory investigations

#### Blood sampling and handling

A blood sample was drawn between 07.00 and 10.00 AM after 12 hours of fasting. Blood samples were centrifuged within 30-45 minutes of collection, and sera were transferred to clean micro-tubes in aliquots, which were kept frozen at -80°C until analysis.

#### Glycemic status, lipid profile and urinary albumin

Fasting serum glucose (FSG), lipid profile including triacylglycerols (TG), total cholesterol (TC), low-density lipoprotein cholesterol (LDL-C), and HDL-C were determined using enzymatic methods. Urinary albumin and creatinine were measured by immunoturbidometric and colorometic methods, respectively. All these tests were done by commercial kits (all from Pars Azmoon, Tehran, Iran) using an auto-analyzer system (Selectra E, Vitalab, Holliston, the Netherlands). As albumin was measured in spot urine samples, urinary albumin to creatinine ratio (ACR) was used to evaluate changes in microalbuminuria. HbA1c was determined using a colorometric method after an initial chromatographic separation (BioSystems, Barcelona, Spain). Serum insulin levels were measured by immunoradiometric assay (IRMA) using a commercial kit (Biosource, Dorest, Belgium) and a γ-counter system (Gamma I, Genesys, Maple Park, USA). Insulin resistance was evaluated by QUICKI calculated as [13]:

QUICKI index:1∕[log (insulin) (μU∕mL)+log (glucose)(mg∕dL)]

#### Circulating 25(OH)D3

Serum 25(OH)D3 concentrations were measured by a high performance liquid chromatography (HPLC) method as described elsewhere [14]. The intra- and inter-assay variations were less than 7% and 9%, respectively. Vitamin D status was defined based on serum concentrations of 25(OH)D as: sufficient ≥50 nmol/L; 27.5 nmol/L ≤ insufficiency < 50 nmol/L; and deficiency < 27.5 nmol/L [15]. It has been recently shown that the vitamin D requirements of at least 97.5% of the population can be met with circulating 25(OH)D concentrations of 50 nmol/L [16]. Serum intact parathyroid hormone (iPTH) (DRG, Marburg, Germany) concentration was determined using enzyme immunoassay (EIA). The intra- and inter-assay variations were less than 3% and 5.5%, respectively.

#### Endothelial biomarkers

Endothelial function was evaluated by determination of serum levels of endothelin-1, E-selectin (both from IBL, Hamburg, Germany) and MMP-9 (e-Bioscience, Vienna, Austria). All ELISA tests were performed with the aid of an automatic plate reader (StatFax 3200, Awareness, Palm City, USA). The intra- and inter-assay variations were less than 8.1% and 8.5% for endothelin-1, 5.4% and 6.0% for E-selectin, and 7.3% and 10.2% for MMP-9, respectively.

#### Body composition analysis

The percentage of body fat was evaluated using bioelectrical impedance analysis (Quadscan 4000 system, Bodystat, Isle of Man, UK).

#### Compliance

All participants were given a pamphlet on "instructions to use dooghs" together with a "doogh consumption table" comprised of 28 empty boxes for each week. Subjects were instructed to mount the table in an exposed place (preferably on the refrigerator) and to tick each box after consuming a doogh with each meal. Moreover, they were asked to bring the empty bottles back on their next visit. Compliance was evaluated by checking the consumption tables, counting the empty bottles, and direct inquiry both on the biweekly visits and weekly follow-up phone calls.

#### Quality control of the product

The composition (including vitamin D_3_) of dooghs was evaluated immediately after production, in the middle and at the end of the storage period, to ensure the stability of the components, especially vitamin D, in the product. The measurements were done at the Maad Laboratory of Foods, Drinks and Cosmetics, accredited by the Deputy of Food and Drug of the Iranian Ministry of Health.

#### Statistical Analyses

Normal distribution of data was assured using Kolmogrov-Smirnov. Data were expressed as mean ± SD. Within- and between-group comparison of data was done using paired *t *test and student *t *test, respectively. To control confounding factors in between-group comparisons, analysis of covariance (ANCOVA) was employed. Correlations between two sets of data were done using the Pearson equation. Categorical variables were compared using chi-square. In this study, *P *value < 0.05 was considered significant. All statistical analyses were done using the statistical package for social sciences (SPSS) for Windows version 11.5 (SPSS Inc., Chicago, IL).

## Results

### General characteristics and dietary intakes

The distribution of age, gender, duration of disease and consumption of statins did not differ significantly between the two groups (Table 1). The average intake of total energy and nutrients (except for vitamin D) also showed no significant within- or between-group difference (data not shown).

**Table 1:** Some selected individual characteristics of the two groups

Variable	PYD (n = 50)	FYD (n = 50)	*P *value
Age (years)	52.4 ± 8.4	52.6 ± 6.3	0.86
Sex (male/female)	19/31	24/26	0.28
Post-menopausal females	18/19	22/24	0.40
Diabetes duration	7 ± 5.2	8.3 ± 4.6	0.18
Smoking no. (%)	1 (2)	5 (10)	0.18
Using statin no. (%)	4 (3.9)	5 (4.9)	0.75

Data presented as mean ± standard deviation or percentage unless stated otherwise

FYD: vitamin D-fortified doogh; PYD: plain doogh

### Anthropometry, body fat mass and blood pressure

The initial values of anthropometric measures did not show any significant differences (Table 2). In the FYD group, compared to the PYD groyp, waist circumference (WC), BMI and FM all decreased significantly by the end of the intervention. However, weight changes did not differ between the two groups. Body FM showed a small but significant increase in the PYD group (*P *= 0.019) and an insignificant decrease in the FYD group. The changes in FM did not differ significantly between the two groups (Table 3). In the FYD group, final values of both systolic (SBP) and diastolic blood pressure (DBP) showed a small but significant decrease compared to the initial values (*P *= 0.007 and *P *= 0.002, respectively), but changes of SBP and DBP did not differ significantly between the two groups (*P *= 0.150 and *P *= 0.090, respectively).

**Table 2:** Comparison of the initial and final values of the variables under study in the two groups

Group	PYD			FYD				
**Variable**	**Before**	**After**	***P* ^i ^**	**Before**	**After**	***P* ^i ^**	***P* ^ii^**	***P* ^iii^**
**Weight**	78.1 ± 13.0	78.8 ± 13.2	0.058	77.6 ± 13.1	77.0 ± 12.8	0.003	0.83	0.49
**Waist (cm)**	101.34 ± 9.1	102.5 ± 9.2	0.035	98.25 ± 9.2	97.5 ± 8.8	0.013	0.090	0.007
**BMI (kg/m^2^)**	30.0 ± 4.2	30.2 ± 4.3	0.079	28.6 ± 4.0	28.4 ± 4.0	0.005	0.09	0.03
**Fat mass (%)**	38.6 ± 9.6	39.8 ± 9.5	0.019	36.2 ± 8.8	34.7 ± 9.4	0.22	0.22	0.01
**SBP (mm Hg)**	128.2 ± 16.6	125.7 ± 6.9	0.22	125.7 ± 14.4	118.5 ± 21.0	0.007	0.42	0.056
**DBP (mm Hg)**	77.8 ± 10.8	77.0 ± 9.3	0.62	78.5 ± 10.3	73.4 ± 8.5	0.002	0.42	0.02
**FSG (mmol/L)**	9.6 ± 2.6	10.0 ± 3.2	0.32	10.5 ± 3.1	9.0 ± 2.8	0.001	0.13	0.10
**Insulin(pmol/L)**	177.8 ± 80.6	204.2 ± 90.3	0.01	141.0 ± 113.2	111.0 ± 82.0	0.002	0.06	0.001
**HbA1c (%)**	8.9 ± 1.6	8.5 ± 1.6	0.087	8.7 ± 1.8	7.8 ± 1.3	0.001	0.10	0.015
**QUICKI**	0.28 ± 0.02	0.27 ± 0.02	0.041	0.29 ± 0.02	0.30 ± 0.02	0.001	0.03	0.001
**TG (mmol/L)**	10.1 ± 4.8	10.1 ± 5.2	0.98	10.3 ± 5.3	8.6 ± 4.5	0.01	0.86	0.001
**HDL-C (mmol/L)**	2.5 ± 0.5	2.4 ± 0.5	0.52	2.6 ± .46	2.8 ± .42	0.043	0.15	0.001
**TC (mmol/L)**	10.1 ± 2.7	10.0 ± 3.0	0.68	10.3 ± 3.1	9.2 ± 1.8	0.001	0.68	0.10
**LDL-C (mmol/L)**	6.15 ± 1.8	6.1 ± 1.8	0.52	5.5 ± 1.7	5.0 ± 1.3	0.003	0.12	0.001
**25(OH)D (nmol/L)**	38.0 ± 22.8	33.4 ± 22.8	0.28	38.5 ± 20.2	72.0 ± 23.5	0.001	0.90	0.001
**iPTH (ng/L)**	55.0 ± 22.5	59.1 ± 18.2	0.28	52.5 ± 24.1	40.1 ± 15.6	0.001	0.12	0.001
**ACR (mg/mmol)**	41.8 ± 106.2	44.1 ± 158.2	0.88	36.2 ± 99.4	31.6 ± 68.9	0.61	0.74	0.40
**Endothelin (μg/L)**	0.80 ± 0.40	0.88 ± 0.41	0.67	0.9 5 ± 0.38	0.75 ± 0.64	0.001	0.50	0.06
**E-selectin (μg/L)**	16.1 ± 6.5	17.1 ± 6.9	0.42	18.1 ± 7.1	14.4 ± 5.7	0.001	0.14	0.035
**MMP-9 (μg/L)**	9.2 ± 5.3	9.6 ± 4.7	0.66	10.6 ± 4.2	8.3 ± 3.7	0.001	0.15	0.12

Data presented as mean ± standard deviation or percentage unless stated otherwise.

*P*^i^: within group difference; *P*^ii^: between-group at the baseline; *P *^iii^: between group after the intervention

FYD: vitamin D-fortified doogh; PYD: plain doogh

**Table 3:** Comparison of changes in biomarkers during treatment with FYD or PYD

	Change in FYD group (n = 50)	Change in PYD group (n = 50)	***P***^**a**^
Waist (cm)	-0.75 ± 2.8	+1.1 ± 3.6	0.005
BMI (kg/m^2^)	-0.17 ± 1.4	+0.25 ± 0.9	0.07
Fat mass (%)	+1.1 ± 3.0	-1.0 ± 5.3	0.23
Systolic BP (mm Hg)	-7.3 ± 18.5	-2.5 ± 14.6	0.15
Diastolic BP (mm Hg)	-3.5 ± 7.8	-0.63 ± 9.3	0.09
FSG (mg/dL)	-26.6 ± 45.6	+6.2 ± 44.8	< 0.0001
Fasting serum insulin (mU/L)	-4.3 ± 9.6	+3.8 ± 10.3	< 0.0001
HbA1c (%)	-0.9 ± 1.4	-0.4 ± 1.7	0.10
Quiccki index	+0.01 ± 0.02	-0.005 ± 0.02	< 0.0001
TG (mg/dL)	-30.3 ± 81.3	+0.12 ± 36.8	0.01
HDL-C (mg/dL)	+2.4 ± 8.3	-0.6 ± 6.7	0.04
TC (mg/dL)	-19.4 ± 34.8	-1.8 ± 31.7	0.009
LDL-C (mg/dL)	-10.7 ± 24.2	-1.5 ± 16.9	0.02
25(OH)D (nmol/L)	+32.6 ± 18.3	-2.7 ± 16.6	< 0.0001
iPTH (pg/ml)	-11.7 ± 20.6	4.3 ± 58.3	0.07
Microalbumin (mg/dl)	-6.3 ± 51.6	0.89 ± 24.8	0.38
Endothelin (ng/mL)	0.03 ± 0.55	0.35 ± 0.63	0.028
E-selectin (ng/mL)	-3.8 ± 7.3	+0.95 ± 8.3	0.003
MMP-9 (ng/mL)	-2.3 ± 3.7	+0.44 ± 7.1	0.02

Data are presented as ± standard deviation

^a^Differences assessed by Student's t-test

FYD, fortified yogurt drink; PYD, plain yogurt drink; BMI indicates body mass index; BP, blood pressure; FSG, fasting serum glucose; HbA1c, glycated hemoglobin; TG, triglyceride; HDL-c high density lipoprotein; TC, total cholesterol; LDL-C, low density cholesterol; 25-OHD,25-hydroxyvitamin D; iPTH, intact parathyroid hormone; MMP-9, matrix metalloproteinase 9.

### Serum 25(OH)D and vitamin D status

As expected, circulating 25(OH)D was raised in the FYD group from baseline (*P *< 0.001) and compared to the PYD group, the increment was significant (*P *< 0.001). While the occurrence of poor vitamin D status (25OHD < 50 nmol/L) did not differ significantly between the two groups at baseline (74.5% versus 77.6%, χ^2 ^= 0.062, *P *= 0.803), the percentage of vitamin D deficiency in the FYD group significantly dropped to only 4.5%, but in the PYG group it increased from 31.4% to 48.8% (*P *< 0.001) (Table 4). The increase in serum 25(OH)D in the FYD group was accompanied by a significant decrement in iPTH (Table 2). Changes of serum iPTH, however, did not differ significantly between the two groups (*P *= 0.07) (Table 3).

**Table 4:** Comparison of the vitamin D status between PYD and FYD groups.

	Deficiency n (%)		Insufficiency n (%)		Sufficiency n (%)	
	**Before**	**After**	**Before**	**After**	**Before**	**After**
**PYD**	16(31.4)	21(48.8)	22(43.1)	14(32.6)	13(25.5)	8(18.6)
**FYD**	13(26.5)	2(4.5)	25(51)	5(4.4)	11(22.4)	37(84.1)
**Total**	29 (28.2)	23 (22.3)	47 (45.6)	19 (18.4)	24 (23.3)	45 (43.7)

*Vitamin D status defined as: Deficiency < 27.5 nmol/L; insufficiency ≥27.5 nmol/L to < 50 nmol/L; and sufficient ≥50 nmol/L. FYD, vitamin D-fortified doogh; PYD: plain dough.

### Glycemic status and lipid profile

Generally, the glycemic status (notably FSG, insulin and QUICKI) in the FYD group, compared to the PYD group, improved significantly. To evaluate the possible effect of the changes of the amount of FM or its distribution on glycemic changes, the associations were re-examined using ANCOVA with both FM and WC together as confounders. The differences in FSG, serum insulin and QUICKI still remained significant (*P *= 0.004, *P *< 0.001 and *P *< 0.001, respectively). Though HbA1c significantly decreased in the FYD group, between-group changes were not significant (*P *= 0.100) (Table 3).

None of the components of the serum lipid profile differed significantly between the groups at baseline. By the end of the intervention, TC, HDL-C and LDL-C did not change in the PYD group but in the FYD group, TC and LDL-C decreased and HDL-C increased significantly (for all *P *< 0.001) (Table 2). Changes in serum concentrations of TC (*P *= 0.009), TG (*P *= 0.01), HDL-C (*P *= 0.044) and LDL-C- (*P *= 0.02) differed significantly between the two groups (Table 3). To evaluate the possible effect of ameliorated insulin resistance on lipid profile, the differences were re-compared using ANCOVA with QUICKI as a confounder. In this case, all of these differences disappeared (TC *P *= 0.093; TG *P *= 0.146; HDL-C *P *= 0.335; and LDL-C *P *= 0.194).

### Biomarkers of endothelial function and urinary albumin to creatinine ratio

Neither endothelial biomarkers nor ACR differed significantly between the two groups at baseline. At the end of the intervention period, however, in the PYD group the changes in these variables were not statistically significant but in the FYD group serum concentrations of endothelin-1, E-selectin and MMP-9 all decreased significantly (Table 2) and between-group differences in their changes were also significant (Table 3). To determine the dependency of changes in the endothelial biomarkers to changes of insulin resistance, the amount or distribution of body fat, or serum 25(OH)D, the differences were re-evaluated using ANCOVA with changes of either QUICKI, FM and WC or 25(OH)D as confounders. The between-group differences in changes of serum endothelin-1 and MMP-9 remained significant even after controlling for changes of QUICKI, FM and WC (*P *= 0.009 and *P *= 0.005, respectively) but disappeared after controlling for serum 25(OH)D (P = 0.066 and *P *= 0.277, respectively). On the contrary, the between-group difference in changes of serum E-selectin remained significant after controlling for 25(OH)D (*P *= 0.011) but disappeared after controlling for QUICKI, FM and WC (*P *= 0.092). The between-group changes in E-selectin (*P *= 0.026), endothelin-1 (*P *= 0.006) and MMP-9 (*P *= 0.008) all remained significant when only QUICKI changes were controlled as a confounder. These changes, however, were not accompanied by a significant decrease in urinary albumin excretion as evaluated by ACR either within or between groups.

## Discussion

The high occurrence of poor vitamin D status in our subjects is comparable with other reports from Iran [10,17]. While the occurrence of vitamin D deficiency and insufficiency did not change after a 12-week intervention period in the PYD group, a significant decrease of poor vitamin D status in the FYD group indicated both the high efficiency of the dosage used and the high bioavailability of vitamin D3 in the Persian yogurt drink, doogh. All participants completed the intervention period and the compliance of the patients was 100%, as judged by checking the consumption tables, follow-up calls, the subjects' reports and, in the FYD group, the expected rise in serum 25(OH)D. However, the persistence of vitamin D deficiency in the FYD group (4.5%) despite a daily intake of 1000 IU vitamin D for 12 weeks can be a subject of considerable argument. Interestingly, those subjects in the FYD group whose vitamin D status remained undesirable after the intervention did not show any appreciable changes in their glycemic and endothelial biomarkers. A similar observation has already been reported [18]. Considering the high prevalence of vitamin D deficiency in Iranian subjects either with or without diabetes [17,19] and assuming the same prevalence of "non-responders" among the whole population, a huge number of people may not benefit from intake of the usually recommended amount of vitamin D. This individual variability may be, at least in part, explained by vitamin D receptor (VDR) polymorphisms. A significant association between VDR variants and susceptibility to several diseases including cancer [20,21], ulcerative colitis [22], metabolic syndrome [23] and both types of diabetes [24,25] has already been reported. The upcoming results from our study group will soon address the role of VDR genotypes on the variation of outcomes after improvement of vitamin D status in subjects with T2D [12].

Significant reductions in BMI, WC and FM following 12 weeks of daily intake of 1000 IU vitamin D via a fortified-doogh, are consistent with our previous report [10]. An inverse association between vitamin D status and adiposity shown in older people [26] was further confirmed in both children [27,28] and adults [29]. From a mechanistic point of view, intracellular calcitriol has been implicated in both enhancing and inhibiting adipogenesis in cell culture [30] probably through peroxisome proliferator-activated receptor-gamma (PPAR-γ) and retinoid × receptor (RXR)-involved pathways [31].

However, some studies have failed to show any additional effect of vitamin D and calcium supplementation during weight loss on body adiposity [32] and in obese children, poor vitamin D status has been suggested as the consequence, rather than the cause, of adiposity [33]. Notwithstanding, in that study obese subjects consumed only 0.25 μg (10 IU)/day of vitamin D [32], a dosage far less than the amount considered sufficient to optimize circulating 25(OH)D [34]. Moreover, weight reduction may mask the possible influence of vitamin D on adipogenesis. To overcome these problems, both an efficient dosage of vitamin D and a weight maintenance intervention were used in the present study.

Poor vitamin D status has been recognized as an independent risk factor for incident arterial hypertension and it is believed that vitamin D supplementation can reduce SBP by 2 to 6 mmHg [35]. However, in this study despite the high occurrence of poor vitamin D status among the participants, no significant effect of regular vitamin D intake on blood pressure (either SBP or DBP) was observed. It should be noted that most of our subjects were normotensive as the blood pressure of only eight (15.4%) and nine (17.6%) subjects in the FYD and PYD groups, respectively, was above 135/85 mmHg. Even re-analysis in the subgroups with above normal BP did not show any significant change. The possible effect of vitamin D on BP merits further research.

Amelioration of the glycemic status of our subjects after a 12-week intervention period further confirms our previous observations [10]. Attenuation, but still persistence, of significant differences after controlling for WC and FM might imply both direct (improvement of insulin secretion and insulin sensitivity) and indirect (decrement of FM and weight) effects of cholecalciferol on glycemic status.

Contrary to a previous report [10], a significant improvement in lipid profile was observed in the FYD group. However, the disappearance of significant between-group changes after controlling for QUICKI implies that the effect of vitamin D3 on the lipid profile may be secondary to its ameliorating effect on glycemic status. Unlike our finding, in a 2-year RCT involving 167 men aged 50 years and older, daily intake of 400 mL low-fat milk fortified with 1,000 mg calcium and 800 IU vitamin D3 did not affect blood lipids [36]. In contrast, daily intake of 1,200 mg calcium and only 400 IU vitamin D in overweight/obese women during a 15-week weight reduction intervention resulted in lower serum LDL-C, compared to those who just had a weight reducing diet [37]. Some of these discrepancies may relate to the baseline blood lipids and 25(OH)D of the subjects. It is noteworthy that our subjects in the present study, compared to the previous one, had lower lipidemic status (especially higher for both TG and TC) so that they might be more responsive to a nutritional intervention. The effect of vitamin D on blood lipids and lipoproteins needs to be elucidated by further precisely controlled clinical trials.

Elevated circulating concentrations of endothelin and E-selectin have been attributed to the boosted production of vasoconstrictors and increased adhesion of and permeability to leukocytes, respectively [38]. On the other hand, a high glucose milieu has been shown to induce over-expression of several matrix metalloproteinases, notably MMP-9, thus promoting matrix degradation, atherogenesis and plaque instability [39]. It has been recently demonstrated that down-regulation of MMP-9 via insulin treatment will result in reduction of intimal lesions in atherosclerosis-prone diabetic apoE (-/-) mice [40]. In the current study, endothelial status, as evaluated by serum concentrations of E-selectin, endothelin-1 and a vascular inflammation marker, MMP-9, were improved after daily intake of 1,000 IU vitamin D via fortified-doogh consumption. This was not, however, accompanied by a significant change in urinary excretion of albumin. Soluble E-selectin together with a coagulation factor, XIIa, has been associated with ten-year macrovascular events in subjects with T2D [41]. Poor vitamin D status-related endothelial dysfunction in diabetes may be partly mediated by nuclear factor κ-B (NF-κB)-linked inflammation [42] and endothelial progenitor cell depletion [43]. Persistence of a significant between-group difference of serum endothelin-1 and MMP-9, but not E-selectin, after controlling for QUICKI, FM and WC implies a direct effect of vitamin D3 on the first two endothelial biomarkers but an indirect effect on E-selectin. It has recently been reported that dyslipidemia and high BMI are related to macroangiopathy while the duration of disease and high HbA1c are associated with microangiopathy [44]. Vitamin D replenishment may, therefore, be considered as an effective preventive measure against development of both macro- and micro-angiopathy by improving lipid profile, BMI and HbA1c. This effect may be comparable with that of the combination of metformin and atrovastatin [45].

Several observational studies have suggested an association between vitamin D deficiency and vascular disease but only a handful of them have examined the effect of vitamin D on endothelial function. In a small pilot study on 34 T2D subjects with vitamin D insufficiency (25OHD < 50 nmol/L), a single, mega dose of vitamin D2 (100,000 IU) resulted in a significant improvement of endothelial function [46]. Monthly intramuscular injection of 300,000 IU vitamin D to subjects with subclinical vitamin D deficiency for 3 months had a favorable effect on endothelial function, as evaluated by brachial artery flow-mediated dilatation (FMD) [9]. In a cross-sectional study, circulating 25(OH)D inversely correlated with MMP-9 and vitamin D status was found to be the only predictor of MMP-9. Following 1-year supplementation of just vitamin D deficient subjects (n = 41), a significant reduction in MMP-9 (-68%) was observed [47]. Our finding of the suppressive effect of vitamin D on serum MMP-9 in subjects with T2D further supports previous reports. Notwithstanding, all these trials have been conducted on rather small sample sizes and in widely disparate settings with different adducts and doses of vitamin D and outcome measures resulting in poor comparability.

In the current study, a significant decrease in serum concentrations of endothelin-1, E-selectin and MMP-9 even after controlling for QUICKI implies the direct effect of vitamin D independent of glycemic status. In accord with this notion, a recent study demonstrated that decreased vascular endothelial cell expression of VDR and 1-α-hydroxylase due to vitamin D deficiency is associated with vascular endothelial dysfunction [48].

However, the limitations of this study must be taken into consideration. Since the study was conducted during the cold seasons, the vitamin D status of our subjects does not reflect its status during the entire year. Moreover, the changes observed after a 12-week intervention cannot necessarily be extrapolated to the long-term effects.

## Conclusions

In conclusion, the present study showed that glycemic status, lipid profile and biomarkers of endothelial functions were all improved following regular vitamin D intake in subjects with T2D but microalbuminuria was not affected significantly. A remarkable rise in circulating 25(OH)D3 following regular intake of fortified doogh indicated the high bioavailability of vitamin D in this Persian yogurt drink. This can be considered by health stakeholders for further fortification programs. Clearly, future randomized and controlled studies with a longer duration and follow up of clinical outcomes are needed to confirm the potentially beneficial effect of vitamin D repletion in patients with T2D.

## Abbreviations

ACR: urinary albumin to creatinine ratio; Ancova: analysis of covariance; BP: blood pressure; BMI: body mass index; CVD: cardiovascular disease; DBP: diastolic blood pressure; EIA: enzyme linked immune assay; EDTA: ethylene diamine tetra-acetate; FM: fat mass; FMD: flow-mediated dilatation; FSG: fasting serum glucose; FYD: fortified yogurt drink; HbA1c: glycated hemoglobin; HDL-C: high density cholesterol; ht: heterozygote mutated; HPLC: high-performance liquid chromatography; hs-CRP: highly sensitive C-reactive protein; 25-OHD: 25-hydroxyvitamin D; iPTH: intact parathyroid hormone; LDL-C: low density lipoprotein cholesterol; MMP-9: matrix metalloproteinase 9; NNFTRI: National Nutrition and Food Technology Research Institute; PYD: plain yogurt drink; QUICKI: Quantitative Insulin Check Index; RCT: randomized clinical trial; SBP: systolic blood pressure; sqFFQ: semi-quantitative Food Frequency Questionnaire; T2D: type 2 diabetes; TG: triacylglycerols; TUMS: Tehran University of Medical Sciences; WC: waist circumference.

## Competing interests

The authors declare that they have no competing interests.

## Authors' contributions

TRN designed and supervised the study, was involved in laboratory analyses and wrote the finalized manuscript. AD and SSB both helped intellectually in finalizing the study design. SSB performed most of the laboratory analyses, wrote the preliminary manuscript and was actively involved in the field work. This study was part of her Ph.D. thesis at TUMS under the supervision of both AD and TRN (grant No: 10533). All statistical analyses were done under the supervision of MRE. AG, together with MZ and SSB, performed HPLC analyses. AK and NS participated in all laboratory investigations. Finally, dietary assessments were performed by HH under AH guidance. All laboratory analyses were performed at the Laboratory of Nutrition Research, NNFTRI. All authors read and approved the final manuscript.

## Pre-publication history

The pre-publication history for this paper can be accessed here:

http://www.biomedcentral.com/1741-7015/9/125/prepub
